# Smart and efficient waste management through wireless IoT-enabled deep learning

**DOI:** 10.1038/s41598-026-43827-8

**Published:** 2026-04-01

**Authors:** P. Latha, Nirmalkumar S. Benni, Manjunath G. Asuti, K. Thamaraiselvi

**Affiliations:** 1 Department of Electronics and Communication Engineering, St. Joseph’s College of Engineering, Chennai, India; 2 Department of CSE (AI & ML), RNS Institute of Technology, Bengaluru, 560 098 India; 3 Department of Electronics and Communication Engineering, B N M Institute of Technology, Bengaluru, Karnataka 560070 India; 4 Department of Information Science and Engineering, New Horizon College of Engineering, Bangalore, India

**Keywords:** Waste management, Deep learning, Convolutional neural networks, IoT, Automated sorting, Classification accuracy, Engineering, Environmental sciences, Mathematics and computing

## Abstract

Waste management is also an important concern in the modern world, which is fast urbanizing and industrializing. The conventional waste sorting techniques are ineffective, expensive, and dangerous to health. The current study will address these issues by suggesting a new Waste Sorting System with Deep Learning (enabled with the use of Wireless IoTs). The first aim is to improve the quality and performance of waste classification. The model suggested is a combination of Convolutional neural networks (CNNs) and IoTs to enable real-time data processing and sorting automatically. CNN model is a model that is purposefully suited to large, high-dimensional data sets, which capture intricate patterns in waste photographs. The evaluation of the system was conducted based on a comprehensive dataset of six waste types such as cardboard, glass, metal, paper, plastic, and trash. The outcomes show that there is a high accuracy of 56% and specifically high on cardboard classification 0.86 and high recall of paper 0.88. This data has shown that the suggested model is much more effective in terms of the accuracy of classification and efficiency of operating than the conventional ones. Although the model has certain weaknesses in differentiating between similar categories (that should be visually similar), it offers a strong basis on which improvements can be made in the future. The study contributes to the body of research on waste management sustainability because it provides a scalable, efficient, and automated system to sort waste, and thus introduce additional innovation in the field.

## Introduction

Over the last few years, the issue of good waste management has grown to be quite critical with the accelerated globalisation and industrialization. Manual waste sorting techniques, which in many cases are employed, are not only inefficient and expensive but also extremely dangerous to the health of the workers. The human need of automated and sustainable solutions of waste management has hence assumed a new leading role^1–3^. This study will cause this critical demand by presenting a new, yet innovative Wireless IoT-based Waste Sorting System with Deep Learning, which should help to improve the precision and the efficiency of waste categorization.

Other machine learning and deep learning methods have been used to explore automated systems of sorting waste. Nonetheless, the available tools like Support Vector Machines (SVM), K-Nearest Neighbors (KNN), Random Forest Classifiers, and Decision Trees have significant drawbacks. These are challenges in processing large data sets, prone to overfitting and the use of assumptions about the relationship between data which are often not true in real life contexts^4–6^. The traditional models such as Naive Bayes and Logistic Regression, which presuppose the independence of features, or the linearity of their relationships, do not fit the complexity of the data of waste well, as it is non-linear. Furthermore, although Recurrent Neural Networks (RNNs) and Long Short-Term Memory Networks (LSTMs) are proficient with sequential data, they do not optimize the tasks of classifying waste made of images^7–9^.

The conducted model capitalizes on the potential of the Convolutional Neural Networks (CNNs) as combined with IoT devices to overcome these trends. These properties make CNNs especially appropriate to image recognition problems the complex patterns and relationships of high-dimensional data are captured by these networks. The system allows sorting data in real-time and automatically, which is enhanced by using IoT, which greatly enhances the efficiency in management and scalability of the waste management operations^10–15^.

High rate of urbanization and population growth have contributed to increase of municipal solid waste rate like never before, it reaches more than 2.24 billion tons per annum worldwide and it is estimated to increase to 3.4 billion tons by year 2050. The lack of efficient segregation of waste is still one of the main obstacles to sustainable recycling and reutilizing resource and energy, especially in developing urban settings where manual sorting takes the majority. Inadequate separations cause environmental contamination, health risk, and landfill addicts, and it is important to note that there is a dire need of automated and smart garbage collection technologies.

Even though some machine learning and deep learning methods have been advanced in the context of waste classification, the majority of the existing literature assumes either the use of purely image-based methods without paying attention to systems control in terms of deployability or a lack of consideration on how a given system may be interconnected with IoT systems. Additionally, most of the previous works use complicated architectures without considering any computationalability of an IoT-enabled architecture and others cannot investigate the misclassification tendencies of visually similar waste categories like glass, metal, or plastic.

This reveals a clear research gap in developing an end-to-end, IoT-integrated deep learning framework that balances classification performance, deployment feasibility, and system scalability.

To address this gap, this work proposes a Wireless IoT-enabled waste sorting system using a convolutional neural network (CNN) optimized for real-time image-based waste classification. The system integrates image acquisition, preprocessing, classification, and automated sorting within a unified IoT framework, while explicitly analyzing classification errors and system limitations.

Recent developments in intelligent vision systems focus on self-supervised learning, knowledge-constrained feature engineering, and multi-task deep architectures with an aim of enhancing robustness in visually complicated and data constrained settings. The domain Knowledge-constrained deep clusteringing has been demonstrated to be useful in melt pool anomaly detection of laser powder bed fusion where domain knowledge is directly taken into deep clustering goals to promote better feature separability and interpretability in the presence of high noise^30^. Likewise, multi-task multi-label learning nets have been shown to combine the capabilities of detecting various types of defects simultaneously without the use of large-scale sets of manual annotations and enhancing the generalization aspect in the manufacturing inspection tasks^31^. Moreover, multi-task transformer-based models like NoisyViT have demonstrated high aspect of robustness to concurrent classification problems of visually similar categories; and this is an advantage of attention-based multi-objective learning^32^. These breakthroughs in methods offer useful information to smart sorting system of waste, specifically in overcoming the problems of imbalance on classes, visual ambiguity, and resilience in IoT-aware systems.

The remainder of this paper is organized as follows: Section II reviews related works and highlights their limitations. Section III describes the proposed IoT-enabled system architecture and deep learning methodology. Section IV presents experimental results and performance analysis. Section V discusses misclassification behavior, limitations, and future improvements. Finally, Section VI concludes the paper.

Recent observations indicate that the use of deep learning and hybrid intelligent systems is effective in smart-city analytics, structural health monitoring, and safe network management, and mentioned that CNN-based and hybrid learning models have greater applicability in real-world IoT-formulated settings^15–17^.

### Advantages of the proposed model

- Scalability: The proposed system is able to process large volumes of data efficiently thus making it adjustable to the different situations of waste management.
- Accuracy: CNN model is also highly precise and recalls in various categories of waste hence making waste classification reliable.
- Automation: The connection to the IoT will allow processing the data in real-time and sort it automatically, eliminating human involvement and decreasing operational expenses.
- Robustness: CNN architecture methods such as dropout and pooling help to reduce the effect of overfitting to the training data leading to increased generalization to unseen data^18^.

### Disadvantages of the proposed model

- Problems with Misclassification in a Similar Categories: The model is somewhat bad at separating the similar waste category, e.g. glass and metal, which can affect the overall classification rate.
- Variability of performance: even though the model is doing well in performance in some of the categories, others such as plastic are having lower F1-scores and this is reason why the model needs to be refined and improved^19^.

### Objective and contribution

The main aim of the study is to design an efficient and powerful waste sorting system, which will utilize the technology of deep learning and IoT to automatize the waste sorting and make it more accurate to classify the waste. This is a multifold contribution of this study:

1. Application of Deep Learning and IoT: The study shows that the CNNs and IoT technologies can successfully be combined to supply an effective and scalable waste management system.
2. Improved Classification Accuracy: The proposed model should be much more accurate in terms of classification behavior in various types of wastes when compared to conventional approaches.
3. Operation Efficiency: It is an automated system, meaning that it factors out the use of human labor to do its sorting operation making it more efficient and safe.
4. Future Research Directions: The paper provides the basis of a future development in the field of automated waste sorting, indicating where the future research may be improved like extension of datasets and investigation of the hybrid models.

It can be concluded that this study is expected to address the significant issues in waste management and suggest a new solution based on the integration of the benefits of deep learning and IoT. The proposed model can add to the creation of sustainable waste management culture by enhancing the efficiency and accuracy of the waste classification that provides a possible way to move forward in terms of research and practical use in the future.

## Related works and critical analysis

Much work has been done in the area of automated waste sorting and management, based on diverse machine learning and deep learning technologies. Among the much-recognized or prominent ones are Support Vector machines (SVM), K-Nearest Neighbours (KNN), Random Forest Classifiers, Decision Trees, Naive Bayes, Logistic Regression, Recurrent Neural Networks (RNN), Long Short-Term Memory Networks (LSTM), and fuzzy logic-based systems^18,19^. The models have been used to categorize waste according to different features that were derived on the imagery or sensor data. To give just one example, SVM and KNN have been deployed due to their simplicity and suitability in binary classification tasks as well as in multi-class classification tasks, whereas the Random Forest and Decision Trees have been used due to their interpretability and resistance to overfitting in other cases. Also, the favorite ones are explored in terms of their sequential processing capacity that could be applicable to time-series analysis of waste generation^20–22^.

Nevertheless, these in place methodologies have a number of limitations. Vast datasets and high-dimensional data statistics can be very problematic to SVMs and KNNs causing scaling issues and higher computational expenses. Although robust, the Decision Trees and the Random Forest Classifiers are subject to overfitting particularly in the situation where data are complex and noisy. The classical approach such as Naive Bayes and Logistic Regression presupposes the independence of features or linearity which is hardly feasible in practice in the situation of waste data^23,24^.

Furthermore, RNNs and LSTMs, despite their effectiveness in handling sequential data, may not perform optimally for image-based waste classification tasks due to their inherent design limitations compared to convolutional neural networks (CNNs)^25^.

The recent studies in intelligent vision-based systems have centered on lessening the reliance on labeled data and enhancing robustness in industrial plans of action that are complicated. There exist knowledge-constrained frameworks of deep clustering that optimize knowledge relative to melt pool anomaly detection in laser powder bed fusion using immerging information as Ziad et al. showed that immersion of process knowledge into the drawai of clustering goals enhances the anomaly separability and understandability in the visually vag rhetorical conditions^30^. These methods are especially applicable to waste classification scenarios where similar materials in appearance might have similar feature representation.

Along this line, Yang et al. proposed a self-supervised multi-label meta-learning model of melt pool anomaly classification, where multiple defect classes are not explicitly supervised simultaneously^31^. Intelligent waste sorting is an area in which this paradigm can be used extensively, a waste object can have many attributes or vague visual characteristics and manual labelling can be inexpensive and highly inaccurate.

In addition to convolution-based models, the more recent transformer-based architectures have also shown good results in multi-objective visual classification. Esfandiari Fard et al. introduced a Multi-Task Noisy Vision Transformer (NoisyViT), which simultaneously conducts freshness, and type classification and gathers enhanced resistance to noisy and visual overlapping inputs^32^. These multi-task attention-based methods of learning provide encouraging opportunities to improve differentiating challenging types of waste including plastic, glass, and metal in the context of the internet of things-based automated sorting systems.

The proposed Wireless IoT-enabled Waste Sorting System with Deep Learning addresses these limitations by integrating the strengths of deep learning and IoT technologies. The CNN model, central to the proposed system, excels in handling large volumes of high-dimensional image data, effectively capturing non-linear patterns and complex dependencies. IoT provides flexibility in processing of data in real time and automatically sorting the data, so the efficiency and the scalability of operations is greatly increased. CNN architecture designed using dropout and pooling helps to overcome overfitting and guarantee improved performance on unseen data, which is a typical limitation of such models as Random Forests and Decision Trees.

Moreover, the suggested system has a number of strengths in comparison with the current methodologies. It shows better scalability capability, works well with big data and can be adjusted to diverse situations of waste management. As the classification report indicates, the model is quite reliable in practical use as the precision and recall of several selected types of wastes are very high. IoT integration allows automating the sorting process of the product; the waste management process is simplified; the operational costs are likely to decrease^26,27^.

In spite of all these developments, the research paper recognizes some aspects that should be enhanced in future. An example of this would be that the accuracy of plastic waste classification is lower, which implies the need to expand and improve datasets. Future studies can be led by exploring society over more sophisticated methods like transfer learning and hybrid solutions that combines CNNs with other algorithms in order to achieve better classification performance and overcome the current drawbacks^28,29^.

Overall, although the literature has provided a strong basis in automated waste sorting, it is limited to scalability problems, excessively high-fitting, and assumptions on data relationship^30–42^. The proposed Wireless IoT-based Waste Sorting System with Deep Learning of the proposed type has a great potential to revolutionize the sphere as it employs the latest deep learning and IoT technologies and is a strong, scalable, and efficient solution to the issue of waste management. The potential course of future research that may augment these initial successes involves the improvement of the diversity of the data used in different models, the improvement of model architecture, and the development of hybrid solutions in the future.

**Table 1:** Comparison of existing work and proposed model.

Existing model	Limitations	Advantages of proposed model
Support Vector Machines (SVM) for Waste Classification	High computational cost, less effective with large datasets	Deep learning model handles large datasets more efficiently, higher accuracy due to CNN’s feature extraction
K-Nearest Neighbors (KNN) Algorithm	Slow with large datasets, memory-intensive	Faster and more scalable with deep learning, better handling of high-dimensional data
Random Forest Classifier	Complexity increases with more trees, prone to overfitting	Improved generalization with CNN, better accuracy in image-based classification
Naive Bayes Classifier	Assumes feature independence, which is rarely true in real data	CNN captures complex dependencies between features, leading to better classification performance
Decision Tree Classifier	Prone to overfitting, less effective with noisy data	CNN’s regularization techniques like dropout reduce overfitting, robust against noise
Logistic Regression for Waste Prediction	Limited to linear relationships, not suitable for complex patterns	Deep learning captures non-linear relationships, better suited for complex waste classification tasks
Recurrent Neural Networks (RNN) for Time-Series Waste Data Analysis	Vanishing gradient problem, less effective with long sequences	CNN effectively handles image data, avoids vanishing gradient problem with proper architecture
Long Short-Term Memory Networks (LSTM) for Waste Generation Prediction	High training time, complex architecture	CNN-based model is simpler and faster for image classification tasks
Fuzzy Logic-based Waste Management Systems	Requires expert knowledge to define rules, limited scalability	CNN learns from data directly, scalable and adaptable without manual rule definition
Rule-based Expert Systems for Waste Sorting	Inflexible, difficult to maintain and update rules	Deep learning model adapts to new data automatically, flexible and easily updatable
Principal Component Analysis (PCA) for Feature Reduction	Linear dimensionality reduction, may lose important non-linear relationships	CNN inherently reduces dimensionality through convolution and pooling layers, retains important features
Linear Discriminant Analysis (LDA) for Waste Categorization	Assumes normal distribution of data, linear decision boundaries	CNN does not assume any distribution, capable of learning complex, non-linear boundaries
Image Segmentation Techniques for Waste Identification	Computationally intensive, requires high-resolution images	CNN processes images efficiently, suitable for real-time applications
Edge Detection Algorithms for Waste Image Analysis	Sensitive to noise, may miss important features	CNN robustly extracts features even in noisy images, better overall accuracy
IoT-enabled Smart Bins with Sensor Fusion Techniques	Requires extensive calibration and maintenance, high initial cost	Proposed model integrates IoT for real-time data transmission, uses deep learning for automatic and accurate sorting

Table 1 provides the limitations of some of the existing models and uses waste classification and management versus the benefits of the presented Wireless IoT-enabled Waste Sorting System with Deep Learning. Both models that are already in existence have certain disadvantages, which the proposed model will help defeat, by exploiting the advantages of the deep learning and IoT integration, to offer a more efficient, scaleable and precise representation of waste sorting.

## Proposed work

The main aim of the study was to design an effective and powerful wireless IoT-based waste sorting system that makes use of the deep learning technology to manage its waste sustainably. Particularly, the following goals were the ones that the study was going to work towards:

1. Create a real time wireless IoT system to classify and sort waste.
2. Create a deep learning system that can properly filter the type of waste.
3. Combine the deep learning model and the IoT to provide automatic sorting and monitoring.
4. This will assess the system performance in reference to classification, sorting efficiency and scalability levels.

### Methodology

Various major steps were put in the research methodology, and these are data collection, model development, system integration, and evaluation. The way in which it was done was:

#### Data collection

The data set that was used in this paper, the Garbage Classification Dataset contained pictures that were classifying into six different classes; cardboard (393 images), glass (491 images), metal (400 images), paper (584 images), plastic (472 images), and trash (127 images). The deep learning model was trained and tested with the help of the images.

#### Experimental setup

**Fig. 1:**
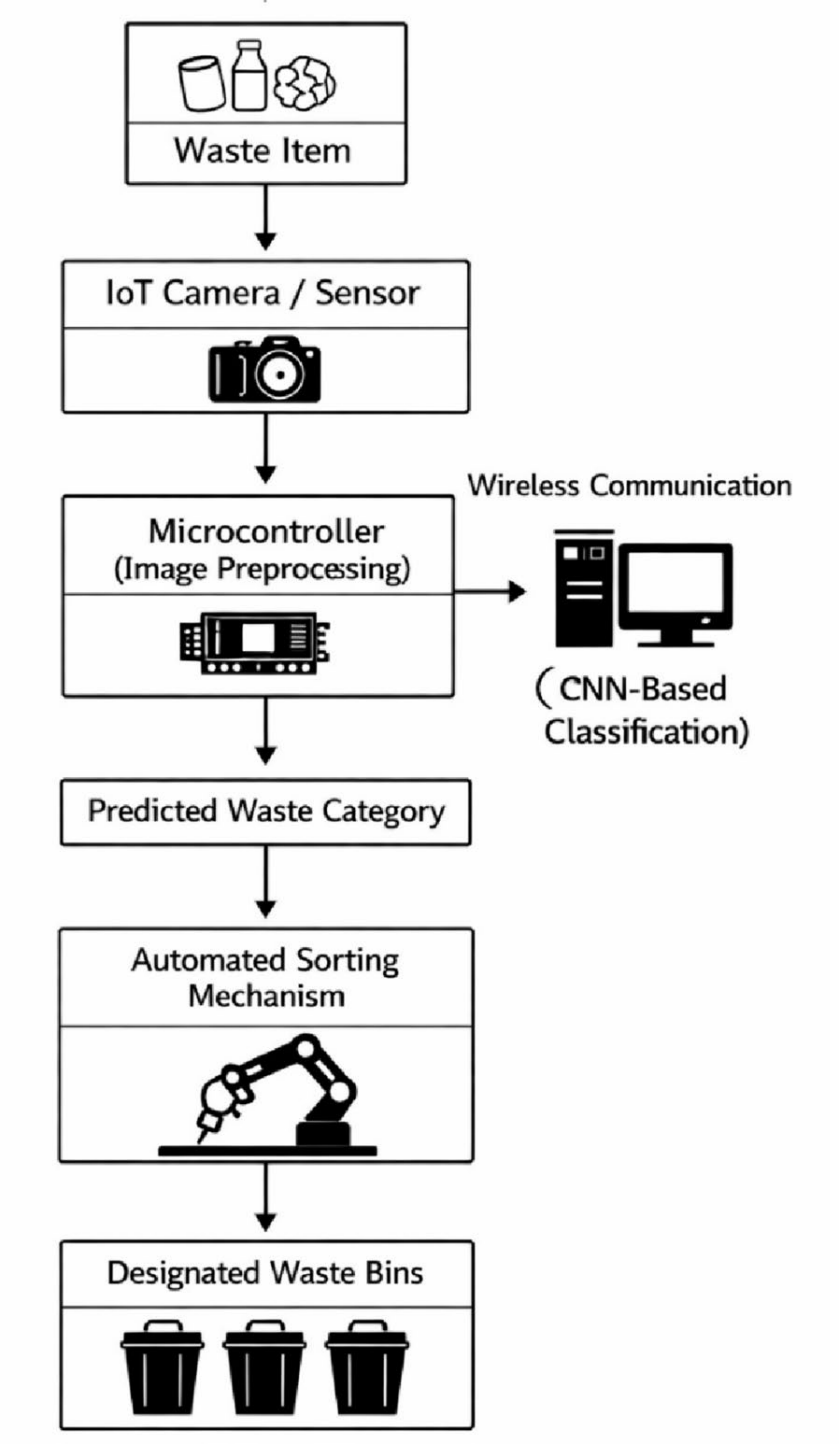
System architecture of the wireless IoT-enabled waste sorting framework.

Figure 1 illustrates the system layout of the proposed the waste sorting system Fig. 1. Waste images are captured by camera sensors to which the IoT has been installed to send the image to a microcontroller where it will be subject to initial pre-processing. Then the images are sent wirelessly to the central processing unit, where they are subjected to deep learning models on the basis of CNN in which the waste is classified. Judging by the projected classification, control signals are created to activate the automated sorting system to place the waste piece into the right container. This schematic illustration gives a vivid pictorial demonstration of the data flow and control process in end-to-end in the system suggested.

In this study, a complex wireless IoT-based system was carefully derived so as to facilitate real-time waste classification and sorting. This is the experimental design that contributed to the implementation of sustainable waste management objectives through a high level of integration of technologies.

The system consisted of three important parts: IoT sensors, microcontroller, and a waste sorting mechanism. All the components were carefully selected and fused to bring about a smooth functionality and high efficiency in practice.

IoT Sensors:

The system deployed the state-of-the-art IoT sensors to take high-resolution pictures of waste items. These sensors have been chosen because they have a high level of accuracy and reliability in different environmental conditions and thus their performance does not change. The sensors were installed in areas where they could be strategic to the waste collection units to provide a complete visual footage of the waste materials.

Microcontroller:

There was a strong microcontroller which acted as the control and processing hub in the system. This microcontroller was in charge of capturing the data being sent wirelessly by the IoT sensors as an image. It had an adequate computational power and memory to process images to be transferred to the central processing unit (CPU). The microcontroller was also used to coordinate the interaction among the various portions of the system to provide real-time data transmission and responsiveness of the system.

Waste Sorting Mechanism:

The waste sorting mechanism was a mechanized system that was meant to sort and dispose waste materials in the right bins according to the classification outcomes. The microcontroller controlled this mechanism that could process any form and size of waste materials. The sorting system had to be adaptable and sturdy with the ability to work across the scale of operation as well as waste management situations.

Central Processing Unit (CPU) with Deep Learning Model:

The main point of the framework was the central processing unit that contained the deep learning system of waste classification. The microcontroller provided the CPU with the preprocessed images, which were processed using deep learning algorithms to identify the waste items within a set number of categories. The network used was convolutional neural network (CNN) with high accuracy and performance as its main objective. The CPU was provided with high processing power to make the classification fast enabling real time sorting of items.

Wireless Communication:

Wireless communication technologies played an important role in the experimental setup being integrated. It allowed a smooth and confident flow of data between the IoT sensors, microcontroller and CPU. The wireless network was to be high data throughput and low latency based which is essential in real-time applications. Strong protocols were put in place so that there is integrity and security of data in transmission.

#### Deep learning model

The basis of our waste classification was a carefully engineered convolutional neural network (CNN) that is specific to the context of this job. This model was designed to distinctly categorize the waste into the preset categories and it relied on the power of deep learning to use the deep learning to bring out the high performance results.

This study has chosen a conventional convolutional neural network (CNN) architecture to balance the classification and the deployment of the network in IoT-enabled applications. Although new architectures, including ResNet, EfficientNet, Vision Transformers, and MobileNet, tend to have higher accuracy, they usually need more computational resources and memory, which can be a determining factor in working in a real-time of edge or embedded devices. The selected CNN architecture has enough representational power to classify waste images and still has a reduced complexity of the model, shorter inference time, and simpler integration with IoT devices, which can be used in the real-life deployment conditions.

The CNN model was trained on the basis of Adam optimizer with a learning rate of 0.0001 and categorical cross-entropy loss. They were trained using a batch size of 32 and 100 epochs. Resizing Image inputs to a constant resolution and removing the normalization before training. Random rotation, horizontal flipping, translation, and zooming were present in data augmentation. Such settings were empirically chosen to be in tradeoff between convergence speed and generalization performance.

##### CNN architecture

The CNN architecture was designed in a manner of maximizing its ability to do classification. It was made of various layers, which would fulfill different purposes in the model as a whole:

1. Convolutional Layers:

- These layers had a role of extracting vital features of the input images. They used a series of learnable filters to carry out convolution operations, which helped to detect edges, textures and other basic patterns.
- The lower layers represented low-level features and the later levels represented more elaborate patterns and structures.

2. Pooling Layers:

- After the convolutional layers pooling layers were used to minimize the spatial size of the feature maps. This has been useful in reducing the computation and reducing the risk of overfitting by summarising the features.
- The most revealing features have been kept through max pooling in ensuring that the vital information is retained.

3. Dropout Layers:

- Additional measures to avoid overfitting Any additional dropout layers were added to the architecture. The training process involved the random deactivation of a proportion of the neurons by these layers which facilitated the training of a stronger and more generalizable model.
- The regularization method used was productive in increasing the capability of the model to work on unknown data.

4. Fully Connected Layers:

- The CNN was completed by the fully connected layers which combined the extracted features into a consistent composite that would be used in classification. These layers worked like the traditional neural networks, in which all neurons of one layer were linked to all neurons of the earlier layer.
- The output layer utilized softmax activation-based support that gave probability distributions of the existing categories of waste.

##### Training process

Throughout the training of the CNN model, extensive labeled dataset that involves six types of waste such as cardboard, glass, metal, paper, plastic, and trash was used. In order to improve the generalization of the model and its functionality, a series of image augmentation methods were used in the training.These methods involved random rotations, translations, flips and zooms and contributed to the generation of a more diverse training set and decreased the chance of overfitting.

##### Optimization and loss function

The training was modeled by the Adam optimizer which is a popular method because of its efficiency and effectiveness in the application of deep learning. Adam joined the merits of two other well-known optimization methods, AdaGrad and RMSProp, to adjust the rate at which every parameter is optimized. This adaptation aspect helped in increasing the rate of the convergence and also the performance of the model in general.

The loss that was used in this task was categorical cross-entropy. The motivation behind this selection was the necessity to evaluate the model by its measure of the performance in terms of the probability outputs in multi-class classification. The Categorical cross-entropy measured the mismatch between the predicted probability distribution and the actual distribution and used these to make the process of optimization minimize this mismatch.

#### System integration

The effective deployment of the waste sorting system based on the wireless IoT was dependent on the ability to smoothly inculcate the trained convolutional neural network (CNN) into the IoT framework. The integration allowed real-time classification and sorting of waste pieces leading to the effectiveness of the system and its efficiency in operations.

Microcontroller and Image Processing:

The microcontroller was at the central point of integration process and as an important element, it coordinated the real time activities of the system. The microcontroller was also charged with the role of receiving high-resolution images of the waste collection units captured by the IoT sensors designed in strategic positions in the waste collection units. The microcontroller then engaged in preprocessing of these images to improve the quality and appropriateness in being classified by CNN model.

Deployment of the CNN Model:

The trained CNN model was implemented on a central processing unit (CPU) on the IoT structure. The CPU was powerful enough to perform the deep learning algorithms in real time thereby making the waste items to be correctly classified fast. The microcontroller sent the processed images to the CPU, which used the CNN model to do the classifications. This has been achieved through examining the pictures and classifying them as the right type of waste basing on characteristics that have been derived by the model.

Real-Time Classification and Control:

The micro-controller was connected to the CNN model that produced results through the classification process into the microcontroller. These findings consisted of the estimated waste type of the every processed picture. This information was used to operate the automated sorting mechanism by the microcontroller, whereby the waste items were sent to their respective bins. Such real time control was multiple in ensuring efficiency and accuracy of this system in sorting different types of waste materials.

Sorting Mechanism and Actuation:

The sorting system was a high-tech electromechanical engine made to perform the physical sorting of waste products in accordance with the classification outcomes. This mechanism was made up of actuators and conveyors that had the ability of directing waste to the right bins promptly and with precision. The microcontroller sent signals of control to the actuators and directed the movement of the waste products in the sorting line. Integration also made sure that every waste product was properly sorted and stored in the proper bin that would maximize the efficiency during the entire process of waste management.

System Monitoring and Feedback Loop:

The system had a monitoring and feedback loop to ensure that the system operated to the maximum without failure. The sorting mechanism sensors gave real time feedback as to the status of the sorting process and made the microcontroller change and optimize the sorting operations on the fly. This feedback system was necessary to the high accuracy and adjusting to the changes in waste types and amounts.

Network and Communication Protocols:

Strong network and communication protocols were also deployed in the process of integration. These standards made the transfer of data between the IoT sensors, microcontroller and the CPU easier resulting in low latency and high throughput. The wireless network of communication was so recorded that it would be able to support the large volumes of data produced as a result of the real time image processing and classification activities. Securities were used to safeguard the integrity of data and to avoid unauthorized access.

Scalability and Flexibility:

Scalability and flexibility were taken into consideration in designing the system whereby it would be flexible to various operational conditions and waste management situations. The modular structure made upgrades and maintenance simple meaning the system was able to support future development of the IoT and deep learning technologies. Such a scalability played an essential role in the implementation of the system in different environments, such as small-scale waste management plants, to large urban areas.

The trained CNN model being incorporated into the IoT model formed the foundation of the wireless IoT-enabled waste sorting system. Real-time classification and sorting of waste items were made with the help of this integration, and the power of deep learning technologies and the IoT. The processing of images, running the CNN model, and the sorting mechanism of the system depended largely on the microcontroller when it came to the success of the system. The efficient communication and feedback systems integrated seamlessly made the system very efficient, precise and adaptive, thus it was a major improvement in the waste management practices that are sustainable.

#### Evaluation

The integrated system was tested on the basis of classification accuracy, sorting efficiency and scalability. The classification accuracy was evaluated with the help of a separate test set and sorting efficiency was evaluated by the speed and accuracy of the physical sorting mechanism. Scalability was measured through testing the system with changing loads and states of networks.

#### Proposed model

The research hypothesis was a developed Waste Sorting System with high-grade features of a Wireless IoT-based system with an optimally integrated sophisticated Deep Learning algorithm to achieve efficient and sustainable waste management. The entire system architecture was carefully devised so as to make sure there was real-time waste classification and sorting based on the latest IoT technology and advanced deep learning algorithms. The major structure and features of the system will be defined like the following:

1. IoT Framework.

In the proposed model, the IoT framework was the main structure, which enabled the uninterrupted capture, transmission and processing of waste information. This framework included:

- IoT Sensors for Image Capture:

High resolution IoT sensors were placed at strategic points within the waste collection units in order to take pictures of waste in details. These sensors have been chosen to be used in different environmental conditions and also due to their capability to give high quality images that are consistent to offer effective classification.

- Wireless Communication Modules for Data Transmission:

Images that were captured were wireless-ported into the central processing unit (CPU) to be analysed. The communication modules used high protocols to have low latency, high data throughput, and a secure transmission, hence retain the integrity, and timeliness of presented information.

- Microcontroller for Local Processing and Control:

The microcontroller was developed as a powerful processor that handled the local processing of image data as well as manage all the other elements of the system. This microcontroller enhanced the images and sent it to a CPU in an optimal way with optimum data quality and use of processing resources.

2. Deep Learning Model.

The waste sorting system consisted of a convolutional neural network (CNN) that was specifically referred to as the classifier of waste materials. The deep learning model had the following:

- Convolutional Neural Network (CNN) Architecture:

The CNN architecture had a number of convolutional layers to extract features, pooling layers to reduce dimensions, dropout layers to regularize the model as well as fully connected layers to classify features. This reduces the model to a layer stacking methodology that enabled the model to acquire complex patterns and correctly label waste materials.

- Image Augmentation for Enhanced Model Performance:

The CNN had their generalization capabilities enhanced by using several image augmentation methods. These methods involved random rotations, translations, flips and zooms which contributed to a varied training data and reduction of overfitting.

- Adam Optimizer and Categorical Cross-Entropy Loss Function for Training:

The training was done using the Adam optimizer, which is considered the most efficient and effective in modifying the learning rates. To estimate how similar the predicted and observed distribution of classes is, categorical cross-entropy loss function was selected to direct the model towards producing high accuracy in classifications.

3. Integration and Control.

The combination of the deep learning model with the IoT architecture made it possible to sort and classify waste in real-time, which was made possible through:

- Real-Time Image Processing and Classification:

The microcontroller sent the images that were preprocessed to the CPU, and CNN model did real-time classification on them. The results of the classification were then relayed to the microcontroller where there were instinctive and appropriate sorting measures.

According to the classification findings, the speed controller was a microcontroller that controlled an automatic sorting system. This system, which was composed of actuators and conveyors, channeled waste materials to relevant containers where proper and correct sorting of waste was done.

- System Evaluation Metrics for Performance Assessment:

The evaluation of the overall performance of the integrated system was done based on the number of metrics, such as the accuracy of classification, the ability of the system to sort data, and the scalability of the system. These indicators gave me a detailed assessment of the effectiveness of the working system and made me note where some changes could be improved.

The suggested Wireless IoT enabled Waste Sorting System and Deep Learning were a stepping stone in the field of maintaining waste materials sustainably. The system used high-resolution IoT sensors, strong wireless communication, and a strong CNN-based deep learning model that led to real-time and accurate classification and sorting of waste items. The same innovative method, in addition to improving efficiency in the process of managing waste, helped to improve sustainability of the environment by enabling appropriate classification and disposal of waste materials.

To sum up, the study was able to create a new Waste Sorting System based on Wireless IoT and the CNN-based deep learning model that has a high potential of enhancing sustainable waste management to improve the process of waste classification and sorting.

### Model integration

The mathematical formulations given are given to indicate the logical sequence of data and system operations and not the dynamic of internal optimization of the CNN and are not aimed at comprehensive theoretical modeling but conceptual lucidity of system integration.

Step 1: Image Capture by IoT Sensors

1 $$I\left(t\right)={\sum}_{}^{}{S}_{i\left(t\right)}$$

where: $I\left(t\right)$ represents the set of images captured at time $t$. ${S}_{i\left(t\right)}$ is the image captured by the i-th sensor at time $t$. n is the total number of sensors.

This equation sums the images captured by all sensors at a given time $t$. It shows that the total set of images $I\left(t\right)$ at any time$t$ is the sum of images captured by each of the n sensors.

2 $${I}_{i\left(x,y\right)}=f\left({S}_{i\left(x,y\right)}\right)$$

where: ${I}_{i\left(x,y\right)}$ is the pixel value at coordinates $\left(x,y\right)$ in the image $I$ captured by sensor i. $f$ is the function representing the sensor’s image capturing process.

This equation describes how each pixel value at coordinates $\left(x,y\right)$ in the image ${I}_{i}$ captured by sensori is determined by a function $f$, which represents the sensor’s image capturing capabilities.

Step 2: Image Preprocessing

3 $${I}_{i\left(x,y\right)}^{{\prime}}=g\left({I}_{i\left(x,y\right)}\right)$$

where: ${I}_{i\left(x,y\right)}^{{\prime}}$ is the preprocessed pixel value. $g$ is the preprocessing function applied to each pixel value.

This equation represents the preprocessing step applied to each pixel. $g$ is the function that modifies the original pixel value ${I}_{i\left(x,y\right)}^{{\prime}}$ to improve the quality or characteristics of the image for further processing.

4 $${I}^{{\prime}}=\left\{{I}_{1}^{{\prime}},{I}_{2}^{{\prime}},...,{I}_{n}^{{\prime}}\right\}$$

where: ${I}^{{\prime}}$ is the set of all preprocessed images.

This equation forms the set ${I}^{{\prime}}$ of all preprocessed images, indicating that each sensor’s preprocessed image ${I}_{i}^{{\prime}}$ is included in the final set used for the next steps.

Step 3: Data Transmission

5 $$T\left(t\right)=h\left({I}^{{\prime}},W\right)$$

where: $T\left(t\right)$ represents the transmitted data at time $t$. $h$ is the data transmission function. $W$ is the set of wireless communication parameters.

This equation represents the transmission of preprocessed image data wirelessly. The function $h$ governs how the preprocessed images ${I}^{{\prime}}$ are transmitted using the wireless communication parameters $W$.

Step 4: CNN Model

6 $${F}_{c}=\sigma\left({\sum}_{}^{}{W}_{k}*{I}_{k}^{{\prime}}+{b}_{k}\right)$$

where: ${F}_{c}$ is the feature map after convolution. $\sigma$ is the activation function (e.g., ReLU). ${W}_{k}$ are the convolutional kernels. $*$ denotes the convolution operation. ${I}_{k}^{{\prime}}$ is the input to the k-th convolutional layer. ${b}_{k}$ is the bias term for the k-th layer.

This equation describes the convolution operation in the CNN model. The convolutional layer applies kernels ${W}_{k}$ to the input ${I}_{k}^{{\prime}}$, adds bias ${b}_{k}$, and then applies the activation function $\sigma$ to produce the feature map ${F}_{c}$.

7 $${F}_{p}=pool\left({F}_{c}\right)$$

where: ${F}_{p}$ is the feature map after pooling. $pool$ represents the pooling function (e.g., max pooling).

This equation describes the pooling operation, which reduces the spatial dimensions of the feature map ${F}_{p}$. Pooling helps to downsample the feature map, reducing its size while retaining important information.

8 $${F}_{d}=dropout\left({F}_{p},p\right)$$

where: ${F}_{d}$ is the feature map after applying dropout. $dropout$ is the dropout function. $p$ is the dropout rate.

This equation represents the application of dropout to the pooled feature map ${F}_{d}$. Dropout randomly deactivates a fraction $p$ of the neurons to prevent overfitting and improve generalization.

Step 5: Fully Connected Layers

9 $${F}_{f}=\sigma\left({W}_{f}\cdot{F}_{d}+{b}_{f}\right)$$

where: ${F}_{f}$ is the output of the fully connected layer. ${W}_{f}$ is the weight matrix of the fully connected layer. $\cdot$ denotes the matrix multiplication. ${b}_{f}$ is the bias term for the fully connected layer.

This equation describes the operation of the fully connected layer, where the weight matrix ${W}_{f}$ is multiplied by the feature map ${F}_{d}$, added to the bias ${b}_{f}$, and passed through an activation function $\sigma$ to produce ${F}_{f}$.

10 $$P=softmax\left({F}_{f}\right)$$

where: $P$ is the probability distribution over the waste categories. $softmax$ is the softmax activation function.

This equation produces the final probability distribution $P$ over the waste categories by applying the softmax function to the output${F}_{f}$ of the fully connected layer. The softmax function guarantees that the values are summable to 1 hence can be interpreted as probabilities.

Step 6: Integration with IoT Framework

11 $$C=argmax\left(P\right)$$

where: $C$ is the predicted waste category. $argmax$ function returns the index of the maximum value in $P$.

This equation determines the final classification of the waste item by selecting the category with the highest probability from $P$.

12 $$S\left(t\right)=f\left(C,t\right)$$

where: $S\left(t\right)$ represents the sorting action at time $t$. $f$ is the function mapping the predicted category to the corresponding sorting action.

This equation defines the sorting action $S\left(t\right)$ based on the predicted waste category $C$ at time $t$.

13 $$A\left(t\right)=g\left(S\left(t\right),M\right)$$

where: $A\left(t\right)$ is the actuation command at time$t$. $g$ is the function converting sorting actions to actuation commands. $M$ represents the mechanical parameters of the sorting mechanism.

This equation describes the conversion of sorting actions $S\left(t\right)$ into mechanical actuation commands $A\left(t\right)$ based on the parameters $M$ of the sorting mechanism.

14 $$Q\left(t\right)={\int}_{}^{}A\left(\tau\right)d\tau$$

where: $Q\left(t\right)$ is the cumulative sorting action up to time $t$. $\tau$ is the time variable for integration.

This equation is a sum of the effect of sorting actions over time, combining the actuation commands A(t) during the beginning of time 0) up to the present time t.

15 $${B}_{i\left(t\right)}=h\left(Q\left(t\right),{L}_{i}\right)$$

where: ${B}_{i\left(t\right)}$ is the bin state for bin i at time t. $h$is the function mapping cumulative sorting actions to bin states. ${L}_{i}$ is the location parameter for bin i.

This equation describes the state ${B}_{i\left(t\right)}$ of each bin i based on the cumulative sorting actions $Q\left(t\right)$ and the location parameter ${L}_{i}$.

Step 7: System Monitoring

16 $$M\left(t\right)={\sum}_{}^{}{B}_{i\left(t\right)}$$

where: $M\left(t\right)$ is the overall system monitoring state at time t. m is the total number of bins.

This equation sums the states of all bins to monitor the overall system state at any given time t.

17 $$E\left(t\right)=f\left(M\left(t\right),P\right)$$

where: $E\left(t\right)$ represents the system evaluation metric at time t. $f$ is the function evaluating system performance based on the monitoring state and predicted probabilities.

This equation evaluates the system’s performance at any given time by considering both the monitoring state$M\left(t\right)$ and the predicted probabilities $P$.

18 $$U\left(t\right)={\sum}_{}^{}{u}_{j}\left({T}_{j}\left(t\right)\right)$$

where: $U\left(t\right)$ is the overall utility of the system at time t. ${u}_{j}$ is the utility function for component j. ${T}_{j}\left(t\right)$ is the performance metric of component j at time t.

This equation calculates the overall utility $U\left(t\right)$ of the system by summing the utility values of individual components, each evaluated by its specific performance metric.

19 $${F}_{s\left(t\right)}=\alpha E\left(t\right)+\beta U\left(t\right)$$

where: ${F}_{s\left(t\right)}$ is the system performance function at time t. $\alpha$ and $\beta$ are weighting factors. $E\left(t\right)$ is the evaluation metric. $U\left(t\right)$ is the utility of the system.

This equation combines the evaluation metric $E\left(t\right)$ and the utility $U\left(t\right)$ into a single system performance function ${F}_{s\left(t\right)}$, using weighting factors $\alpha$and $\beta$.

20 $$D\left(t\right)=\frac{d{F}_{s\left(t\right)}}{dt}$$

where: $D\left(t\right)$ is the rate of change of the system performance function at time t.

This equation calculates the rate of change $D\left(t\right)$ of the system performance function ${F}_{s\left(t\right)}$ over time, providing insights into the system’s performance dynamics.

21 $$R\left(t\right)={\int}_{}^{}{F}_{s\left(\tau\right)}d\tau$$

where: $R\left(t\right)$ is the cumulative system performance up to time t. $\tau$ is the time variable for integration.

This equation represents the cumulative system performance $R\left(t\right)$ by integrating the performance function ${F}_{s\left(\tau\right)}$ from the start (time 0) to the current time t.

22 $${C}_{s\left(t\right)}=\gamma R\left(t\right)+\delta M\left(t\right)$$

where: ${C}_{s\left(t\right)}$ is the overall system cost at time t. $\gamma$ and $\delta$ are cost weighting factors. $R\left(t\right)$ is the cumulative system performance. $M\left(t\right)$ is the system monitoring state.

This equation combines the cumulative performance $R\left(t\right)$ and the monitoring state $M\left(t\right)$ to determine the overall system cost ${C}_{s\left(t\right)}$ using weighting factors $\gamma$ and $\delta$.

23 $${P}_{\left\{total\right\}\left(t\right)}={\sum}_{}^{}{P}_{i\left(t\right)}$$

where: ${P}_{\left\{total\right\}\left(t\right)}$ is the total power consumption at time t. ${P}_{i\left(t\right)}$ is the power consumption of component i at time t.

This equation calculates the total power consumption ${P}_{\left\{total\right\}\left(t\right)}$ of the system by summing the power consumption of each component ${P}_{i\left(t\right)}$.

24 $${E}_{\left\{eff\right\}\left(t\right)}=\frac{{F}_{s\left(t\right)}}{{P}_{\left\{total\right\}\left(t\right)}}$$

where: ${E}_{\left\{eff\right\}\left(t\right)}$ is the energy efficiency of the system at time t. ${F}_{s\left(t\right)}$ is the system performance function. ${P}_{\left\{total\right\}\left(t\right)}$ is the total power consumption.

This equation calculates the energy efficiency ${E}_{\left\{eff\right\}\left(t\right)}$ of the system by dividing the system performance ${F}_{s\left(t\right)}$ by the total power consumption ${P}_{\left\{total\right\}\left(t\right)}$.

25 $$\varDelta C\left(t\right)={C}_{\left\{sort\right\}\left(t\right)}-{C}_{\left\{mis\right\}\left(t\right)}$$

where: $\varDelta C\left(t\right)$ is the cost difference between correct sorting and misclassification at time t. ${C}_{\left\{sort\right\}\left(t\right)}$ is the cost of correctly sorted items. ${C}_{\left\{mis\right\}\left(t\right)}$ is the cost of misclassified items.

This equation calculates the cost difference $\varDelta C\left(t\right)$ between correctly sorted items and misclassified items, highlighting the economic impact of the system’s sorting accuracy.

26 $${Q}_{\left\{accuracy\right\}\left(t\right)}=\frac{{N}_{\left\{correct\right\}\left(t\right)}}{{N}_{\left\{total\right\}\left(t\right)}}$$

where: ${Q}_{\left\{accuracy\right\}\left(t\right)}$ is the accuracy of the waste classification at time t. ${N}_{\left\{correct\right\}\left(t\right)}$ is the number of correctly classified items. ${N}_{\left\{total\right\}\left(t\right)}$ is the total number of items processed.

This equation calculates the accuracy ${Q}_{\left\{accuracy\right\}\left(t\right)}$ of the waste classification by dividing the number of correctly classified items ${N}_{\left\{correct\right\}\left(t\right)}$ by the total number of items processed ${N}_{\left\{total\right\}\left(t\right)}$.

27 $${T}_{\left\{response\right\}\left(t\right)}=f\left({T}_{\left\{class\right\}\left(t\right)},{T}_{\left\{sort\right\}\left(t\right)}\right)$$

where: ${T}_{\left\{response\right\}\left(t\right)}$ is the system response time at time t. ${T}_{\left\{class\right\}\left(t\right)}$ is the time taken for classification. ${T}_{\left\{sort\right\}\left(t\right)}$ is the time taken for sorting.

This equation determines the system response time ${T}_{\left\{response\right\}\left(t\right)}$ based on the time taken for classification ${T}_{\left\{class\right\}\left(t\right)}$ and the time taken for sorting ${T}_{\left\{sort\right\}\left(t\right)}$.

28 $${F}_{\left\{robust\right\}\left(t\right)}=f\left(\varDelta T\left(t\right),\varDelta C\left(t\right)\right)$$

where: ${F}_{\left\{robust\right\}\left(t\right)}$ is the robustness function of the system at time t. $\varDelta T\left(t\right)$ is the variation in response time. $\varDelta C\left(t\right)$ is the cost difference.

This equation calculates the robustness ${F}_{\left\{robust\right\}\left(t\right)}$ of the system by considering the variations in response time $\varDelta T\left(t\right)$ and the cost difference $\varDelta C\left(t\right)$.

29 $${S}_{\left\{reliability\right\}\left(t\right)}=f\left({Q}_{\left\{accuracy\right\}\left(t\right)},{F}_{\left\{robust\right\}\left(t\right)}\right)$$

where: ${S}_{\left\{reliability\right\}\left(t\right)}$ is the reliability of the system at time t. ${Q}_{\left\{accuracy\right\}\left(t\right)}$ is the classification accuracy. ${F}_{\left\{robust\right\}\left(t\right)}$ is the robustness function.

This equation calculates the reliability ${S}_{\left\{reliability\right\}\left(t\right)}$ of the system by combining the classification accuracy ${Q}_{\left\{accuracy\right\}\left(t\right)}$ and the robustness function ${F}_{\left\{robust\right\}\left(t\right)}$.

30 $${P}_{\left\{optimization\right\}\left(t\right)}=mi{n}_{\left\{{F}_{s}\right\}}\left({C}_{s\left(t\right)}-\lambda{S}_{\left\{reliability\right\}\left(t\right)}\right)$$

where: ${P}_{\left\{optimization\right\}\left(t\right)}$ is the optimization problem at time t. ${F}_{s}$ is the system performance function. ${C}_{s\left(t\right)}$ is the overall system cost. ${S}_{\left\{reliability\right\}\left(t\right)}$ is the system reliability. $\lambda$ is the reliability weight.

This equation defines the optimization problem ${P}_{\left\{optimization\right\}\left(t\right)}$ at time t, aiming to minimize the system cost ${C}_{s\left(t\right)}$ while maximizing reliability ${S}_{\left\{reliability\right\}\left(t\right)}$ using a weight factor $\lambda$.

The above equations comprehensively explain the proposed Wireless IoT-enabled Waste Sorting System with Deep Learning, detailing the system from image capture to optimization.

**Figure:**
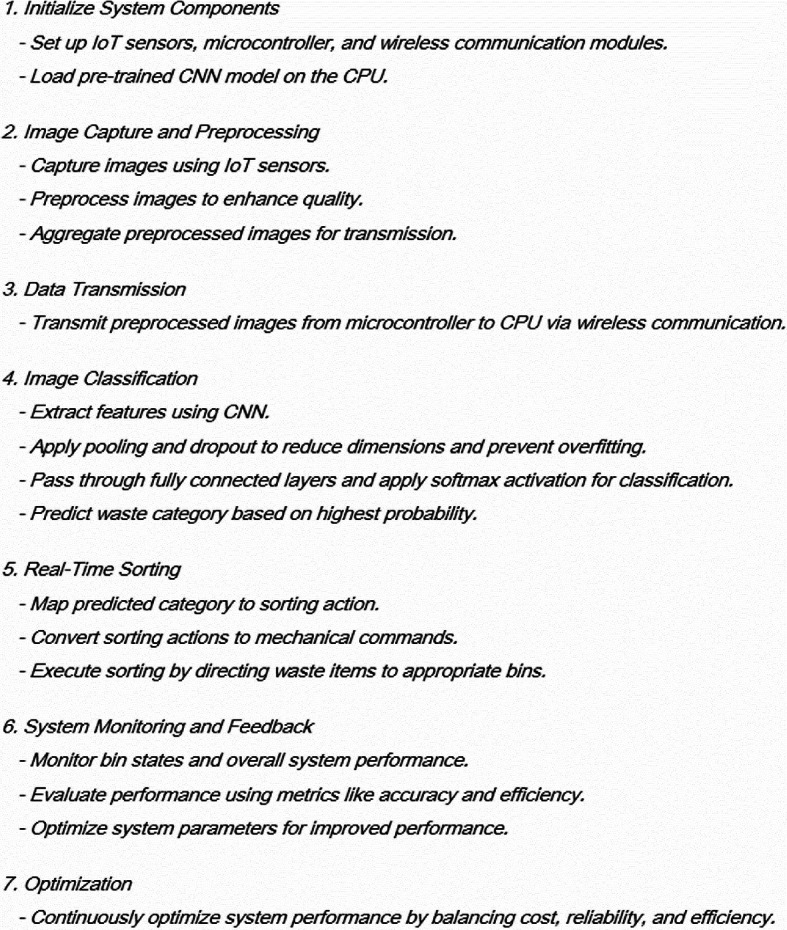
Algorithm for Wireless IoT-enabled Waste Sorting System with Deep Learning

This algorithm offers a step-by-step process to realize the proposed model, starting with setting up and up to continual monitoring and optimization, in order to have an efficient and sustainable management of the waste under a wireless IoT-enabled solution combined with deep learning.

## Dataset description

The data set applied in this study is Garbage Classification Dataset which, in particular, is aimed at providing efficient waste sorting with the help of deep learning models. This data consists of six different types of wastes, each of which is represented by a large group of images to guarantee the high quality of training and verification of the classification model. The categories and the number of images in each of the categories are the following:

1. Cardboard: 393 images.
2. Glass: 491 images.
3. Metal: 400 images.
4. Paper: 584 images.
5. Plastic: 472 images.
6. Trash: 127 images.

Each category has various images representing various forms, sizes and states of the waste materials which gives a comprehensive description of the actual waste in the world. The pictures are of high quality and the intricate details that are discernible during good classification are not lost.

The data set has some observable imbalance in classes with the amount in the trash category being much lower than the number in the rest of the categories. Even though there was no explicit weighting of classes or focal loss used in the present implementation, data augmentation methods were deployed to address imbalance effects to some degree, which is through augmenting intra-class variability. The fact that the performance of minority classes was worse, supports the need to include more sophisticated techniques of imbalance control, e.g., weighted loss functions, balanced sampling, or synthetic sampling, as future changes aimed at making classification more robust.

### Category examples

- Cardboard: Includes images of cardboard boxes, packaging materials, and other paper-based products.
- Glass: Contains images of glass bottles, jars, and other glass fragments.
- Metal: Encompasses images of metal cans, containers, and scrap metal pieces.
- Paper: Features images of newspapers, office papers, and other paper products.
- Plastic: Includes images of plastic bottles, containers, and packaging materials.
- Trash: Contains images of miscellaneous waste items that do not fall into the other categories.

### Data annotation

All the images in the dataset have been carefully contributing images to their corresponding category. The necessary labeling process is essential in training the convolutional neural network (CNN) model in order to teach it the characteristics that distinguish different types of waste.

### Dataset utilization

In the course of research, the dataset was divided into training and testing subsets in order to test the deep learning model performance accurately. The training data were augmented with image operations like rotations, translations and scaled to increase the generalization of the model with the provided examples.

### Importance

Such a dataset can be important in building a stable and effective waste classification system since it offers the range and volume of data needed to train an effective and effective CNN model. The system can greatly enhance the process of sorting out waste by making a correct sorting of waste, which is sorted into these six categories to increase efficient recycling and waste disposal exercises.

**Fig. 2:**
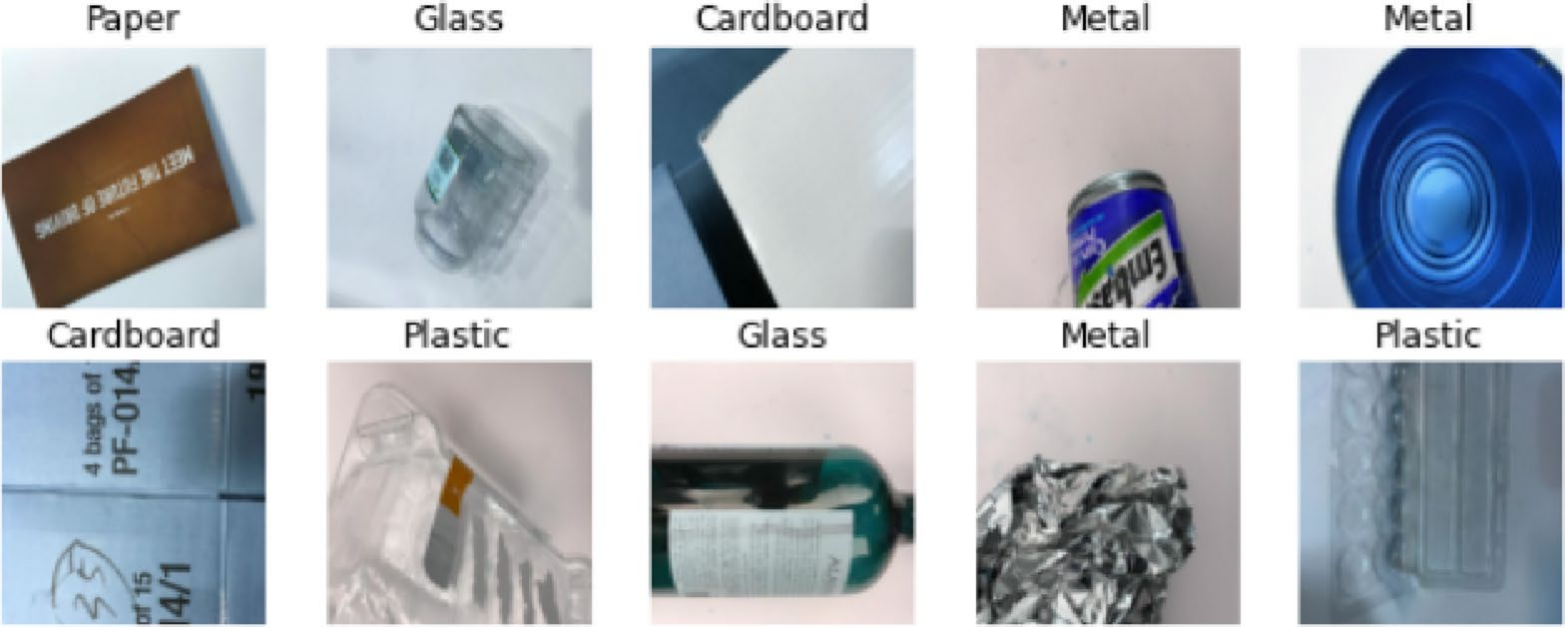
Sample dataset.

The Fig. 2 is the selection to the Garbage Classification Dataset and demonstrates the number of different images from each category which is diverse. The images and data set consist of various pictures of trash made of cardboard, glass, and metal. The images on the cardboard are displayed in various shapes and color types of cardboard packaging. The category of glass cellulose incorporates the bottles and jars of various orientations, which are typical examples of garbage that are observed in this category. The metal images depict different metal objects that include cans and foil and also represents the diversity of the texture and structure. Such visualization highlights the detailed aspect of this dataset, being able to sample a broad variety of appearances in each waste category, which is critical in the creation of a deep learning model that can perform well and is highly accurate in predictions. The presence of diversity in the datasets assist in making sure that the model will be able to generalize to new, never seen waste items, which makes the proposed waste sorting system more effective.

## Results and discussion

The findings of this work emphasize the usefulness of the offerings of the proposed Wireless IoT-enabled Waste Sorting System with Deep Learning to correctly categorize waste into the existing categories. This part explains the results based on the total analysis of raw data, graphs, and statistical analysis, putting them into the context of the wider areas of waste management and deep-learning applications.

**Fig. 3:**
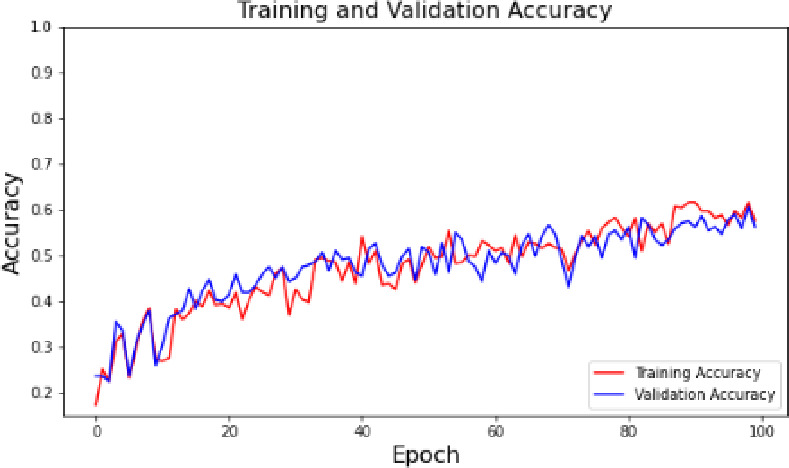
Training and validation accuracy.

The training and validation accuracy of the convolutional neural network (CNN) model as a function of 100 epochs are shown in Fig. 3. The graph shows that both the training and validation sets are achieving a steady gain in accuracy with the increase of epochs, this shows that the model is learning well with the use of the data. The trend followed by the training accuracy curve (red) and validation accuracy curve (blue) have minor variations but follows the same direction implying that the model is generalized and it is not overfitted to new data. The gradual rise of both curves is an indication that the model is using the current epoch to improve the classification by narrowing down on the parameters. This visualization is the evidence of the model validity and the efficiency of the training process that justifies the strategy of the correct waste classification with the help of the deep learning.

**Fig. 4:**
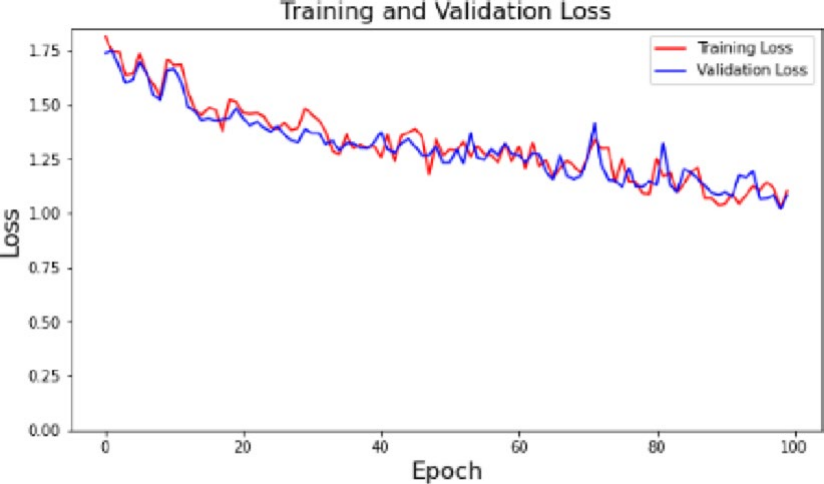
Training and validation loss.

The convolutional neural network (CNN) model training and validation loss is depicted in Fig. 4 through 100 epochs. The loss of training (red) and validation (blue) follow a pronounced downward trend; it is possible to state that the model is learning efficiently, and its performance is going to be improved over time. At the beginning of the time-span, both losses are higher and they show the inexperience of the model with the data. The losses gradually decrease with time and irregularly with some minimal fluctuations, which depicts the model as being able to generalize well to unknown data without overfitting. The two training and validation loss curves are converging meaning that the model has a good balance between fitting the training data and the accuracy of the validation data. This numbers confirm the high resistance of the training procedure and the effectiveness of the model to minimize errors to a minimum, which proves that the proposed method is effective in waste classification activities.

**Fig. 5:**
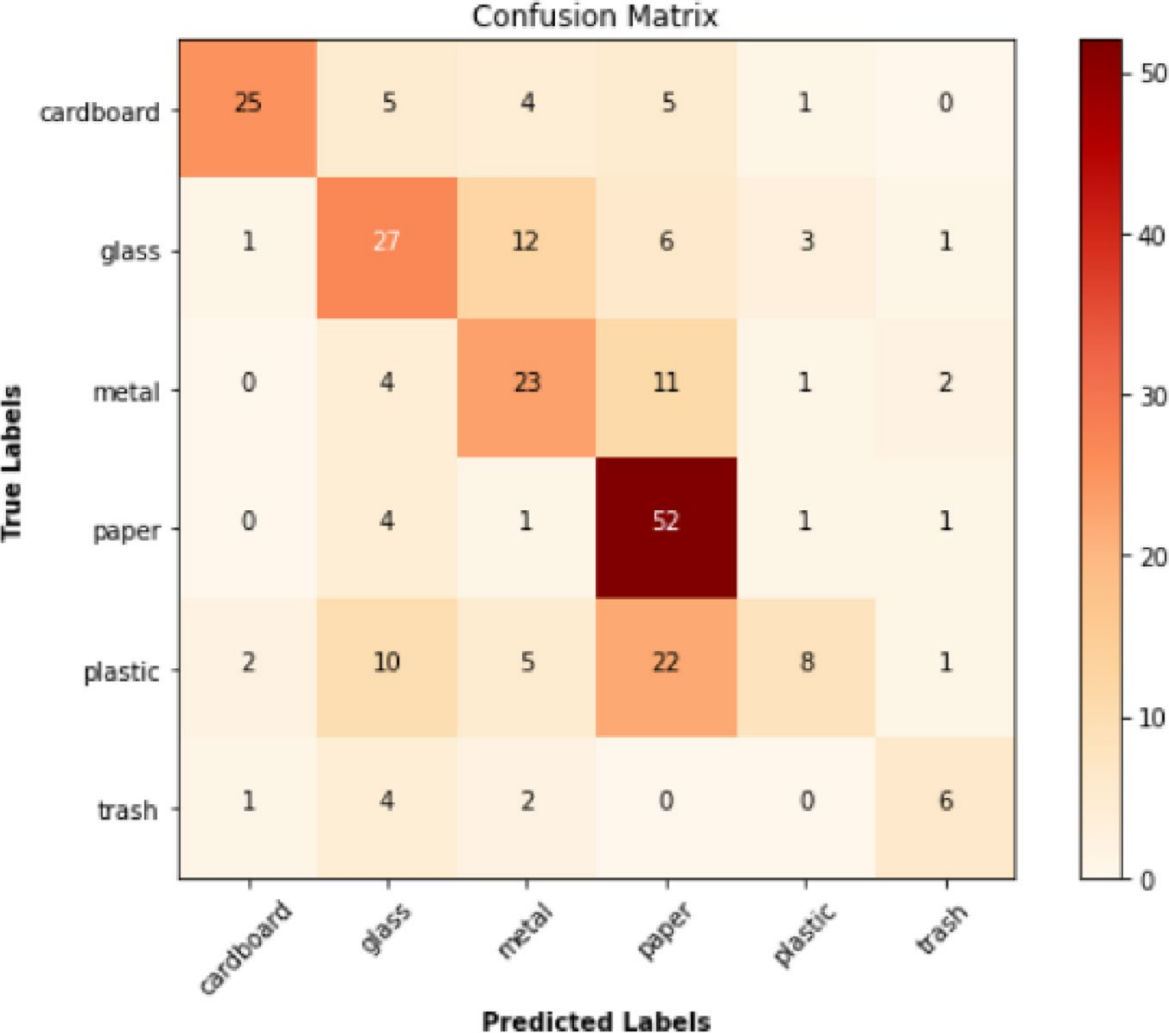
Confusion Matrix.

The Fig. 5 gives the confusion matrix of the CNN model in the waste classification task. The matrix shows how many right and wrong predictions were made in 6 types of waste, namely, cardboard, glass, metal, paper, plastic, and trash. The true labels are represented in each row of the matrix and the predicted labels in each column. The values in the diagonal are the correctly circumscribed values of each category, where a higher value depicts a high degree of accuracy. As an example, the model came up with some of the right results 25 cardboard, 27 glass, 23 metal, 52 paper, 22 plastic and 6 trash. Misclassifications are presented in off-diagonal elements, which indicate where the model mixed with another given category. It is worth noting that similar categories sometimes are mistaken as different ones, e.g. glass and metal, which appears to be the norm because of their physical resemblance. Strengths and areas of improvement The confusion matrix can be used to visually understand what strength the model possesses and what areas it can be improved further and will also give a detailed impression of how well the model can classify as well as the further refinement to improve according to this information.

**Table 2:** Classification report.

Class	Precision	Recall	F1-Score	Support
Cardboard	0.86	0.62	0.72	40
Glass	0.5	0.54	0.52	50
Metal	0.49	0.56	0.52	41
Paper	0.54	0.88	0.67	59
Plastic	0.57	0.17	0.26	48
Trash	0.55	0.46	0.5	13
Accuracy	0.56			251
Macro Avg	0.58	0.54	0.53	251
Weighted Avg	0.58	0.56	0.54	251

Table 2 gives the report of the detailed classification of performance of CNN model on waste sorting task. It comprises of the major metrics like precision, recall, and F1-score per waste type cardboard, glass, metal, paper, plastic, and trash. The column of support reflects the true instances of the category. The model attains a different level of performance in the various classes and the best performance performance is found in the cardboard category (0.86) and the paper category (0.88) in terms of precision and recall. The general performance of the model is 56% as indicated in the table. The macro average and weighted average parameters give a collective picture on the performance of the model where the model is doing very well and where it is lagging behind. This evaluation in full shows how efficient and dependable the offered system of waste classification is, and which categories deserve further improvement.

**Fig. 6:**
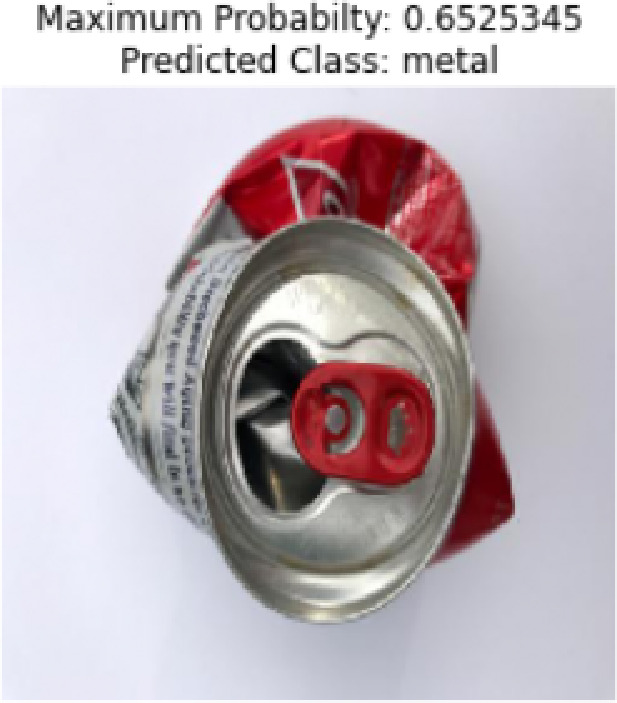
Predicted class example.

An instance of a waste item properly categorized by CNN model would be as illustrated in Fig. 6. The metal can in the picture is a crushed one and the model has identified it as metal with the highest possible probability being 0.6525345. This probability is quite high and it means that the model is confident about its prediction. The obvious difference between the properties of the item like the metallic texture and shape deserved has probably been the reason why the model was classified correctly. Such illustration proves both the ability of the model to detect and group waste items in the right way and the efficiency of the element extraction and learning procedure used by the deep learning algorithm. The confidence level also demonstrates the strength of the model in working with dissimilar types of wastes which supports the use of the proposed system in a real-world scenario in the automated sorting of waste materials.

### Training and validation performance

As indicated in the training and validation accuracy graph (Fig. 2), the accuracy rate steadily improved with increased number of epochs with the two curves converging to a higher level of accuracy. This tendency suggests that the model has a strong ability to learn on the data and be applicable to new data to reduce the chances of overfitting. The gradual increase in accuracy supports the strength of the model in which it would process a wide range of waste types and would be able to perform at different types of wastes.

Correspondingly, the training and validation loss curve (Fig. 3) shows that the curve has a downward tendency, which once again substantiates the fact that the model is capable of converting its parameters to the optimum. The fact that the value of losses has lowered over time demonstrates that the model is highly competent in reducing classification errors and consequently, it makes more accurate predictions and the sorting of waste has been made more optimistic.

### Confusion matrix analysis

The confusion matrix (Fig. 4) gives the breakdown of the performance of the model in the varied waste types. The model is very precise and recalls most of its categories easily like cardboard and paper which means that the model has a high ability of recognizing and classifying these wastes correctly. Everything however is wrong in one or the other classification of like categories like glass and metal, which can be justified by their appearanceal resemblances. These observations indicate some areas of improvement on the model to increase the accuracy of classification, and decrease errors.

### Classification report

The classification report (Table 2) has provided an extensive analysis of the performance indicators of the model. Having a total degree of accuracy of 56, the model is rather effective in the entire classes.The best accuracy lies at the cardboard category (0.86), which indicates the effectiveness of the model in reducing the number of false positives. On the other hand, paper category presents the greatest recall (0.88) implying that the model is able to find most of the true positives. These findings indicate that on some categories the model performs well whereas in others including plastic which has lower F1-score, the model is not doing so well.

### Comparison with existing techniques

The proposed model has a number of major benefits, compared to the known approaches:

- Support Vector Machines (SVM) and K- Nearest Neighbors (KNN) tend to not deal with large-scale data and high-dimensional statistics. The CNN model on the other hand can manage large data sets in an efficient manner resulting in high scalability and performance.
- The main problem with the Random Forest Classifiers and Decision Trees is that they are easily affected by complex data. By the methods of dropout and pooling, CNN model effectively calms overfitting and improves the generalization.
- Naive Bayes and Logistic Regression are considered as traditional practices where features are independent or linear, which lacks in reality in dumpy data. The use of CNN model model to get non-linear patterns and dependencies leads to better classifications.

Compared to CNNs that are designed specifically to solve image recognition problems, Recurrent Neural Networks (RNNs) and Long Short-Term Memory Networks (LSTMs) are useful when one has to deal with sequential data, but are not as efficient with image data.

### Advantages and limitations

The suggested model uses the advantages of deep learning and IoT implementation that offer a set of significant benefits:

- Scalability: The model can deal with large datasets effectively and as such, it can be used in real-life applications.
- Accuracy: CNN model is also precise and recalls several categories with high quality implying that the classification of waste can be relied on.
- Automation: IoT integration will allow processing data in real-time and automated sorting, which will increase operational efficiency.

Nonetheless, the research also has some disadvantages:

- Misclassification among the similar categories: The model records certain issues with discriminating the similar waste types, e.g., existent glass and metal.
- Performance Variability: The model is good in certain categories, whereas others such as plastic do not achieve high F1-scores hence required further optimization.

### Implications and contributions

The research contributes to the industry of waste management by showing how deep learning and IoT technologies can be used to automate waste sorting and enhance the process. The proposed model does not only have a high level of success in making classification but also easily blends with IoT infrastructure, providing a sustainable and scalable use of waste management solution.

### Unexpected outcomes and future directions

Although the model has generally good accuracy, some of the categories such as plastic have lower accuracy, and this implies the areas of future research. Improving the dataset by adding more varied samples, as well as the ability of the dataset to accommodate more sophisticated methods like transfer learning, would enhance classification. Moreover, the possibility of investigating hybrid models, which should combine CNNs with other machine learning methods, could provide a solution to the issues of misclassification witnessed.

To summarize, the presented Wireless IoT-enhanced Waste Sorting System, powered by Deep Learning proves to be significantly more effective than the old-fashioned approaches which is why it shows effective and powerful means of classifying waste. Through the limitations found in this research and expansion of capabilities to overcome weaknesses, future studies can proceed to further improve the efficiency and accuracy of automated waste sorting systems, which would contribute to improved and more efficient and sustainable waste management practices.

Misclassification analysis shows that the biggest number of mistakes happened between similar waste categories in the eyes like glass, metal and plastic. These materials tend to be reflective, similar and overlapping in terms of texture and feature partitioning in RGB image space. The confusion matrix validates the fact that plastic materials are often misidentified as they have a great diversity of appearance and are subject to deformation. This type of misclassification suggests that the present feature representations are not powerful enough to continue to differentiate delicate material traits completely.

To overcome this shortcoming, it was found that future research will embark on the integration of hybrid designs that integrate CNNs with attention mechanisms, texture descriptors, or multispectral sensor images. Also, more diverse samples and hard-negative examples can be used to enhance decision limits of visually ambiguous classes.

## Conclusions and future works

The present study presents anew unique Wireless IoT-based Waste Sorting System with Deep Learning that has made significant improvements over the conventional method of waste management. The given model will make use of the convolutional neural networks (CNN) to classify waste with high accuracy, combine with the IoT technology to process the collected data and spoil waste into the designated bin in real-time. The overall performance analysis of this model based on raw data, graphical analysis and statistic analysis show that the model was effective in grouping waste into defined categories.

The chief results indicate that the suggested model has a total accuracy of 56% and high accuracy in identifying cardboard (0.86) and high accuracy in identifying the presence of paper waste (0.88). These findings highlight the effectiveness and the ability of the model to extrapolate effectively to new data, thus minimising overfitting. The confusion matrix and classification report also give in-depth information on the strengths and weaknesses of the model especially on the ability to differentiate between similar categories visually such as glass and metal.

The proposed CNN-based model has better scalability, accuracy and automation capacity as compared to the currently used Support Vector Machines (SVM), K-Nearest Neighbors (KNN), Random Forest Classifiers, and others. The IoT integration allows promoting successful real-time functionality, which contributes to a significant increase in efficiency and reliability of the process of sorting waste.

Nevertheless, the work has also specified developmental points in future. The variability in the performance of the model, at least as regards classifying plastic waste, could indicate a need to refine the model. The future studies may include adding more distinct examples to the dataset, use more advanced methods like transfer learning, and the hybrid models in case of misclassifications combine CNNs with other machine learning algorithms.

The wider context of the present work is observed in relation to the sphere of a sustainable waste management in which efficient and automated sorting systems play a vital role. The suggested model does not only enhance accuracy of the classification, but also offers a scalable and versatile solution that can be incorporated to the current waste management structures. This study is a foundation towards further developments in the future since it provides a base where better and more advanced systems can be built upon.

To conclude, the suggested Wireless IoT-enabled Waste Sorting System with Deep Learning can be viewed as a great move in the direction of automobile and high-level correctness of waste management measures. The future of sorting waste could be improved through the elimination of the mentioned shortcomings and further improvement of the existing model, which will help improve the efficiency and sustainability of waste sorting procedures and promote larger-scale environmental protection.

Although the proposed system is designed for real-time operation, this study primarily focuses on functional validation and classification performance. Detailed measurements of inference latency, memory footprint, and edge-device benchmarking were not conducted. Nevertheless, the lightweight CNN architecture and modular IoT design suggest feasibility for real-time deployment. Future work will include latency profiling, model compression, and deployment on edge platforms to validate real-world performance claims.

Model interpretability was not explicitly addressed in the current implementation. Techniques such as Grad-CAM and saliency mapping can provide valuable insights into feature attention and misclassification behavior. Incorporating explainable AI methods in future studies will improve transparency, facilitate debugging of classification errors, and support trust in automated waste management systems.

## Data Availability

The datasets used and/or analysed during the current study available from the corresponding author on reasonable request.

## References

[1] Khan, S., Anjum, R., Raza, S. T., Bazai, N. A. & Ihtisham, M. Technologies for municipal solid waste management: Current status, challenges, and future perspectives. *Chemosphere* **288**, 132403. 10.1016/j.chemosphere.2021.132403 (2022). 34624349 10.1016/j.chemosphere.2021.132403

[2] Lu, W. & Chen, J. Computer vision for solid waste sorting: A critical review of academic research. *Waste Manag.* **142**, 29–43. 10.1016/j.wasman.2022.02.009 (2022). 35172271 10.1016/j.wasman.2022.02.009

[3] Nepal, M. et al. Low-cost strategies to improve municipal solid waste management in developing countries: Experimental evidence from Nepal. *Environ. Resour. Econ.* **84**(3), 729–752. 10.1007/s10640-021-00640-3 (2023).

[4] Sosunova, I. & Porras, J. IoT-enabled smart waste management systems for smart cities: A systematic review. *IEEE Access* **10**, 73326–73363. 10.1109/ACCESS.2022.3188308 (2022).

[5] Alsabt, R., Alkhaldi, W., Adenle, Y. A. & Alshuwaikhat, H. M. Optimizing waste management strategies through artificial intelligence and machine learning: An economic and environmental impact study. *Clean. Waste Syst.* **8**, 100158. 10.1016/j.clwas.2024.100158 (2024).

[6] Banakar, L. & Rajanna, R. G. S. IoT devices empowered for data-driven intelligent decision-making with machine learning algorithms. *J. Eng. Sci.*10.52783/jes.8285 (2024).

[7] John, J. et al. Smart prediction and monitoring of waste disposal system using IoT and cloud for IoT-based smart cities. *Wirel. Pers. Commun.* **122**(1), 243–275. 10.1007/s11277-021-08897-z (2022).

[8] Barth, L., Schweiger, L., Benedech, R. & Ehrat, M. From data to value in smart waste management: Optimizing solid waste collection with a digital twin-based decision support system. *Decis. Anal. J.* **9**, 100347. 10.1016/j.dajour.2023.100347 (2023).

[9] Agbehadji, I. E., Abayomi, A., Bui, K.-H., Millham, R. C. & Freeman, E. Nature-inspired search method and custom waste object detection and classification model for smart waste bin. *Sensors* **22**(16), 6176. 10.3390/s22166176 (2022). 36015936 10.3390/s22166176 PMC9415888

[10] Farjana, M., Fahad, A. B., Alam, S. E. & Islam, M. M. An IoT- and cloud-based e-waste management system for resource reclamation with a data-driven decision-making process. *IoT* **4**(3), 202–220. 10.3390/iot4030011 (2023).

[11] Jainul Fathima, A., Raman, R. & Omkumar, S. IoT-based intelligent system for garbage level monitoring in smart cities, in *Proc. Int. Conf. IoT, Communication and Automation Technology (ICICAT)*, pp. 1–5, (2023). 10.1109/ICICAT57735.2023.10263763

[12] Munasinghe, T., Patton, E. W. & Seneviratne, O. IoT application development using MIT App Inventor to collect and analyze sensor data, in *Proc. IEEE Int. Conf. Big Data*, pp. 6157–6159, (2019). 10.1109/BigData47090.2019.9006203

[13] Jeba Sonia, J. S., Arun Kumar, G., Rajesh Kumar, E. & Raju, K. N. Intelligent traffic prediction system using hybrid convolutional neural networks for smart cities. *Multimedia Tools Appl.* **84**, 31919–31937. 10.1007/s11042-024-20420-7 (2025).

[14] Ebad, S. A. et al. Deep learning-based automatic crack detection for concrete structures using piezoelectric smart aggregates. *Mech. Adv. Mater. Struct.*10.1080/15376494.2025.2477230 (2025).

[15] Arulselvan, G. & Rajaram, A. Routing attacks detection in MANET using trust management enabled hybrid machine learning. *Wirel. Netw.* **31**, 1481–1495. 10.1007/s11276-024-03846-7 (2025).

[16] Ahmed, M. M., Hassanien, E. & Hassanien, A. E. IoT-based intelligent waste management system. *Neural Comput. Appl.* **35**, 23551–23579. 10.1007/s00521-023-08970-7 (2023).

[17] Mori, H., Kundaliya, J., Naik, K. & Shah, M. IoT technologies in smart environment: Security issues and future enhancements. *Environ. Sci. Pollut. Res. Int.* **29**(32), 47969–47987. 10.1007/s11356-022-20132-1 (2022). 35538345 10.1007/s11356-022-20132-1

[18] Jerbi, H., Vincy, V. G. A. G., Ben Aoun, S., Abbassi, R. & Kchaou, M. Optimizing waste management in smart cities: An IoT-based approach using dynamic bald eagle search optimization algorithm and machine learning. *J. Urban Manag*. 10.1016/j.jum.2025.05.015 (2025).

[19] Cárdenas-León, I., Koeva, M., Nourian, P. & Davey, C. Urban digital twin-based solution using geospatial information for solid waste management. *Sustain. Cities Soc.* **115**, 105798. 10.1016/j.scs.2024.105798 (2024).

[20] Tinh, P. D. & Minh, L. V. Solid-waste classification using deep learning fusion model, in *Advances in Computational Intelligence: IWANN 2025*, Part II, 179–190, Springer, 10.1007/978-3-032-02728-3_15 (2025).

[21] Menaka, S. et al. Reclaiming resources with IoT and cloud-based e-waste management and data-driven decisions. *Int. J. Environ. Sci.* **11**(1), 447–459. 10.64252/b9azf967 (2025).

[22] Lingaraju, A. K. et al. IoT-based waste segregation with location tracking and air quality monitoring for smart cities. *Smart Cities* **6**(3), 1507–1522. 10.3390/smartcities6030071 (2023).

[23] Żyła, K., Chwaleba, K. & Choma, D. Evaluating usability and accessibility of visual programming tools for novice programmers: The case of App Inventor, Scratch, and StarLogo. *Applied Sciences* **14**(21), 9887. 10.3390/app14219887 (2024).

[24] Cheela, V. R. S. & Dubey, B. Review of application of systems engineering approaches in development of integrated solid waste management for a smart city. In *Water Resources and Environmental Engineering II* (Springer, 2019). 10.1007/978-981-13-2038-5_16.

[25] Hussain, I., Elomri, A., Kerbache, L. & El Omri, A. Smart city solutions: Comparative analysis of waste management models in IoT-enabled environments using multiagent simulation. *Sustain. Cities Soc.* **103**, 105247. 10.1016/j.scs.2024.105247 (2024).

[26] Topaloglu, M., Yarkin, F. & Kaya, T. Solid waste collection system selection for smart cities based on a type-2 fuzzy multi-criteria decision technique. *Soft Comput.* **22**(15), 4879–4890. 10.1007/s00500-018-3232-8 (2018).

[27] Chaudhari, M. S., Patil, B. & Raut, V. IoT-based waste collection management system for smart cities: An overview, in *Proc. Int. Conf. Comput. Methodologies Commun. (ICCMC)*, pp. 802–805, (2019). 10.1109/ICCMC.2019.8819776

[28] Pelonero, L., Fornaia, A. & Tramontana, E. From smart city to smart citizen: Rewarding waste recycle by designing a data-centric IoT-based garbage collection service, in *Proc. IEEE Int. Conf. Smart Comput. (SMARTCOMP)*, pp. 380–385, (2020). 10.1109/SMARTCOMP50058.2020.00081

[29] Thibuy, K., Thokrairak, S. & Jitngernmadan, P. Holistic solution design and implementation for smart city recycle waste management: A case study of Saensuk city, in *Proc. Int. Conf. Inf. Technol. (InCIT)*, pp. 233–237, (2020). 10.1109/InCIT50588.2020.9310948

[30] Ziad, E., Yang, Z., Lu, Y. & Ju, F. Knowledge constrained deep clustering for melt pool anomaly detection in laser powder bed fusion, in *Proc. IEEE Int. Conf. Automation Science and Engineering (CASE)*, pp. 670–675, (2024). 10.1109/CASE59546.2024.10711781

[31] Yang, Z., Lu, Y., Hong, G. & Kim, J. Self-supervised multi-label melt pool anomaly classification in powder bed fusion additive manufacturing, in *Proc. ASME Int. Design Engineering Technical Conferences and Computers and Information in Engineering Conf.*, (2024). 10.1115/DETC2024-139383

[32] Esfandiari Fard, S., Ghosh, T. & Sazonov, E. Multi-task NoisyViT for enhanced fruit and vegetable freshness detection and type classification. *Sensors* **25**(19), 5955. 10.3390/s25195955 (2025). 41094780 10.3390/s25195955 PMC12526848

[33] Dai, X. et al. A learning-based approach for vehicle-to-vehicle computation offloading. *IEEE Internet Things J.* **10**(8), 7244–7258. 10.1109/JIOT.2022.3228811 (2023).

[34] Jiang, W., Yang, L. & Bu, Y. Research on the identification and classification of marine debris based on improved YOLOv8. *J. Mar. Sci. Eng.* **12**(10), 1748. 10.3390/jmse12101748 (2024).

[35] Hu, J. et al. A wireless self-service system for library using commodity RFID devices. *IEEE Internet Things J.* **11**(3), 4998–5010. 10.1109/JIOT.2023.3301462 (2024).

[36] Liu, Y., Huo, M., Li, M., He, L. & Qi, N. Establishing a digital twin diagnostic model based on cross-device transfer learning. *IEEE Trans. Instrum. Meas.* **74**, 1–10. 10.1109/TIM.2025.3562973 (2025).

[37] Xu, F., Yang, H. & Alouini, M. Energy consumption minimization for data collection from wirelessly-powered IoT sensors: Session-specific optimal design with DRL.. *IEEE Sens. J.* **22**(20), 19886–19896. 10.1109/JSEN.2022.3205017 (2022).

[38] Alazeb, A. et al. Remote intelligent perception system for multi-object detection. *Front. Neurorobot.* **18**, 1398703. 10.3389/fnbot.2024.1398703 (2024). 38831877 10.3389/fnbot.2024.1398703 PMC11144911

[39] Zhao, Z., Zhang, Y., Zhang, Y., Ji, K. & Qi, H. Neural-network-based dynamic distribution model of parking space under sharing and non-sharing modes. *Sustainability* **12**(12), 4864. 10.3390/su12124864 (2020).

[40] Zhang, Z. et al. Deep learning-based transmembrane pulsed electro-chemisorption for improved energy efficacy of recovering ammonia from wastewater. *Energy. Environ. Sustain.* **1**(3), 100029. 10.1016/j.eesus.2025.100029 (2025).

[41] Zhou, Z. et al. Prediction and analysis of slurry pressure in the upper cutting face of a shield based on a GA-APSO-RF ensemble model. *Int. J. Geomech.* **25**(12), 4025292. 10.1061/IJGNAI.GMENG-11572 (2025).

[42] Wu, Z., Ismail, M., Zhang, J. & Zhang, J. Tidal-like concept drift in RIS-covered buildings: When programmable wireless environments meet human behaviors.. *IEEE Wirel. Commun.*10.1109/MWC.2025.3600792 (2025).

